# Vitamin D3 Priming of Dendritic Cells Shifts Human Neutrophil-Dependent Th17 Cell Development to Regulatory T Cells

**DOI:** 10.3389/fimmu.2022.872665

**Published:** 2022-07-07

**Authors:** Florianne M. J. Hafkamp, Esther W. M. Taanman-Kueter, Toni M. M. van Capel, Tom Groot Kormelink, Esther C. de Jong

**Affiliations:** Department of Experimental Immunology, Amsterdam Institute for Infection and Immunity, Amsterdam University Medical Center, University of Amsterdam, Amsterdam, Netherlands

**Keywords:** vitamin D3, dendritic cells, neutrophils, Th17 cells, Treg, autoimmune disorders

## Abstract

Vitamin D3 (VD3) is a potential adjuvant for use in tolerogenic vaccine formulations that target dendritic cells (DCs) for the treatment of chronic inflammatory disorders, e.g., autoimmune diseases. These disorders are often associated with enhanced activity of IL-17-producing T helper 17 (Th17) cells which develop in a DC-driven and neutrophil-dependent fashion. Here, we investigated the effect of VD3 on *Candida albicans*-specific human T-cell differentiation, since *C. albicans* is a model pathogen for Th17 cell development. VD3 priming of DCs restricted neutrophil-dependent Th17 cell development and neutrophil-independent Th1 cell formation from naive CD4^+^ T cells. In line with this, the production of Th1/Th17-polarizing cytokines IL-12 and IL-23 by DCs was reduced by VD3 priming. Development of both FoxP3^+^CD127_low_CD25^+^ Tregs and IL-10-producing T cells was significantly enhanced in VD3-primed conditions, even in the presence of neutrophils. ICOS^+^ Tregs, major IL-10 producers, CD69^+^FoxP3^+^, and TIGIT^+^FoxP3^+^ Tregs were significantly induced by VD3 priming as well. Our data support the potential use of VD3 as an adjuvant to induce tolerance in the treatment of autoimmune disorders, including those in which neutrophils are involved in pathogenesis, since we show that Treg development is enhanced by VD3 even in the presence of neutrophils, while Th17 cell development is restricted.

## Introduction

Interleukin 17 (IL-17)-producing Th17 cells constitute a pathogenic cell type associated with chronic inflammatory disorders, including psoriasis, rheumatoid arthritis, and neutrophilic asthma (1–3). On the other hand, Th17 cells are crucial players in the defense against pathogens such as the fungus *Candida albicans*, as inborn IL-17 or IL-17R deficiency results in chronic severe mucocutaneous candidiasis (4). Pathogen-primed dendritic cells (DCs) do not induce Th17 cells from naive human T cells *in vitro*, while they are able to induce Th17 cells from human memory T cells (5). Ligation of nucleotide-binding oligomerization domain 2 by pathogen-derived muramyl dipeptide programs DCs to produce elevated levels of IL-1β and IL-23 (5), cytokines alongside IL-21 and transforming growth factor β (TGF-β) important for induction and propagation of human Th17 cells (6–8). In DC-driven Th17 cell development from naive T cells, neutrophils are crucial accessory cells. We demonstrated that neutrophil elastase cleaves DC-derived CXCL8 of 77aa [CXCL8 (77)] at the N-terminus into the short 72 aa form CXCL8 (72) and only the shorter form potently induces human Th17 cell differentiation (9). Among others, Th17 cells enhance the recruitment of neutrophils to the inflammatory site, enforcing an uncontrolled inflammatory loop (2, 10). Therefore, restriction of Th17 cell development by tipping the balance toward regulatory T cells (Tregs) to dampen chronic inflammation could be beneficial in the treatment of various autoimmune diseases.

The vitamin D3 (VD3) metabolite calcitriol is a known potent immunomodulatory agent capable of tolerance induction to restore immune balance in autoimmune disease and other chronic inflammatory disorders (11). VD3 either directly polarizes human T cells toward FoxP3^+^ Tregs (12, 13) or exerts its tolerogenic effect *via* DCs that promote the development of FoxP3^+^ and/or IL-10-producing Tregs (11, 14–18). Tregs exist in various forms and subsets, and for specific Treg types, IL-10 production is crucial for their suppressive function, such as inducible costimulatory (ICOS)^+^ Tregs (19, 20). VD3 has been shown to enhance IL-10 production by DCs (15, 18, 21). Furthermore, exposure of DCs to VD3 reduces their capability to polarize T cells to T helper 1 (Th1) cells (17, 22), corresponding with decreased IL-12 release (17, 22–25). VD3 suppresses the transcription of IL-12 subunits p35 and p40 through downregulation of nuclear factor kappa B (NF-κB) activity (24). The p40 subunit is shared with IL-23, a cytokine that supports Th17 cell development (2). Hence, VD3 is a potent anti-inflammatory compound that could shift the development of pro-inflammatory Th1/Th17 cells to Treg development for the treatment of autoimmune diseases.

In addition to the well-known Treg-inducing effects of VD3, it was shown in mice that VD3 reduces Th17 cell development (26, 27). Reduced expression of IL-23p19 and IL-6 in mice treated with calcitriol correlates with suppressed Th17 cell formation (26, 27). Also, an *in-vitro* human study demonstrated that VD3-treated DCs induce fewer IL-17^+^ T cells from total CD4^+^ T cells than control DCs (28). The question remains how VD3 influences DC-driven Th17 cell development from human naive T cells, which is a neutrophil-dependent polarization due to the requirement for cleaved CXCL8 (9). Here, we investigated how VD3 priming of DCs affects antigen-specific DC-driven T-cell differentiation in an established co-culture model of DCs, naive T cells, and neutrophils, known to skew Th17 cell induction (9). We show that VD3 priming of DCs impedes neutrophil-dependent Th17 cell development and reduces Th1 cell formation while increasing Treg development, also under these Th17 cell-polarizing conditions. The induced Tregs are functional suppressor cells and they express various functional Treg markers. Strikingly, we observe an additive effect of neutrophils in Treg development induced by VD3-primed DCs, and we speculate that neutrophils may contribute to Treg induction independently of VD3. Taken together, given the potency of VD3 to induce immune tolerance even in the presence of neutrophils, VD3 is a potent adjuvant for the treatment of chronic inflammatory disorders, including autoimmune diseases with excessive and pathogenic neutrophil infiltration (29–31).

## Materials and Methods

### Cell Isolation

Blood from healthy donors was collected after informed consent in heparin tubes (Greiner Bio-One, Alphen a/d Rijn, The Netherlands) for peripheral blood mononuclear cell (PBMC) and neutrophil isolation by density gradient centrifugation on Lymphoprep (*d =* 1.077 ± 0.001 g/ml; Axis-Shield, Oslo, Norway). Subsequently, PBMCs were separated into monocyte and peripheral blood lymphocyte (PBL) fractions by density gradient centrifugation on Percoll (GE Healthcare, Hoevelaken, The Netherlands). Monocyte-derived DCs were generated from the monocyte fraction, as described previously (9), and the purity of DCs was 95% or higher. CD4^+^CD45RA^+^ naive cells were subsequently purified by negative selection and CD4^+^CD45RO^+^ memory cells by positive selection from the PBL fraction by magnetic cell separation, as described previously (9). The purity of obtained naive T cells always exceeded 98%, and T cells were stored in liquid nitrogen until use in co-culture. Neutrophils were isolated fresh on the day of co-culture initiation from the erythrocyte pellet of Lymphoprep density centrifugation, as described previously (9). The purity of neutrophils was always more than 98%. The purity of all cell types was analyzed by flow cytometry and is shown in **Supplementary Figure 1**.

### Hyphae Generation

Cultures of *C. albicans* (clinical isolate) were maintained at the Department of Experimental Microbiology, Amsterdam University Medical Center, Amsterdam. Yeast particles were resuspended in Iscove’s modified Dulbecco’s medium (IMDM; Thermo Scientific, Gibco, Waltham, MA, USA) supplemented with 10% heat-inactivated fetal bovine serum (Sigma-Aldrich, St. Louis, MO, USA) w/o gentamycin and plated at 10,000 particles per well on flat-bottom 96-well plates (Costar, Corning Inc, Corning, NY, USA). For the scraping of hyphae, 60,000 particles were plated in 24-well plates (Costar). (Pseudo-)hyphae were formed during 4 h of incubation at 37°C, 5% CO_2_, followed by heat killing for 2 h at 70°C.

### Co-Cultures

On day 6 of DC generation, immature DCs were primed for 2 h with 2.5 µM of VD3 metabolite calcitriol 1,25(OH)2D3 purchased from Sigma-Aldrich in IMDM/5% FCS prior to harvesting. Titrations of VD3 were done to determine the optimal concentration for use, and we primed for only 2 h given that 30% of cholecalciferol, a highly lipophilic compound, was taken up within 1 h of culture of Caco-2 cells (32). DCs were extensively washed prior to use in culture since VD3 has been demonstrated to have a profound direct effect on T cells (33). Co-cultures were done in IMDM supplemented with 5% heat-inactivated human serum (Lonza, Verviers, Belgium) and gentamycin (86 μg/ml, Duchefa Biochemie B.V., Haarlem, The Netherlands). Culturing was done initially in 96-well *C. albicans* hyphae-coated plates with 50,000 DCs and 50,000 autologous naive CD4^+^ T cells, in the presence or absence of 100,000 autologous neutrophils. These culture conditions for Th17 cell development were optimized previously (9). In indicated experiments, recombinant human IL-23 (R&D Systems, Minneapolis, MN, USA) or epacadostat (Selleck Chemicals, Houston, TX, USA) was used (epacadostat pretreatment of DCs for 30 min at 37°C, no washing). Cells were transferred after 4 days to 48-well plates (Costar) and refreshed with a medium containing recombinant human IL-2 (Novartis AG, Basel, Switzerland) at a final concentration of 10 U/ml every 2 days. Dependent on the growth of the culture, cells were transferred to 24-well plates 2 to 4 days later and cultured until resting on days 11–13 of culture when cytokine production was determined either by flow cytometry (IL-17 and IFN-γ) or by ELISA for IL-10, and cells were stained for Treg markers by flow cytometry. T cells (100,000) were restimulated with anti-CD3 (clone 1XE, 0.15 µg/ml) and anti-CD28 (15E8, 1 µg/ml) (both purchased from Sanquin, Amsterdam, The Netherlands) in IMDM 5% heat-inactivated human serum on flat-bottom 96-well plates, and 24-h supernatants were collected to measure IL-10 by ELISA. To assess the stimulatory capacity of DCs, 30,000 DCs were co-cultured with 50,000 human naive CD4^+^ T cells in the presence or absence of 60,000 neutrophils (2:1 ratio neutrophil:DC) on *C. albicans* hyphae-coated plates. On day 5 of culture, the percentage of proliferating T cells was assessed by measuring the incorporation of EdU into dividing cells by flow cytometry using the Click-iT™ EdU kit (C10424, Invitrogen, Waltham, MA, USA) according to the manufacturer’s instructions.

### T Cell Suppressor Assay

T cells that were co-cultured for 8 days with either control or VD3-primed DCs with or without neutrophils (“test cells”) were incubated for 2 h at 37°C with 100 µg/ml of mitomycin C (Sigma), to prevent expansion, and then harvested, extensively washed, and counted. Autologous memory T cells were labeled with 5,6-carboxyfluorescein diacetate succinimidyl ester (0.5 mM; Molecular Probes, Eugene, OR, USA) and subsequently used as bystander target cells. Test cells (50,000) were co-cultured in a round-bottom 96-well plate (Costar) with 25,000 target cells and 500 beads/well Dynabeads™ CD3/CD28 (Gibco). After 3 to 4 days, the proliferation of the target T cells was determined by flow cytometry.

### DC Maturation

After 2 h of priming of immature DCs in a 24-well plate with calcitriol (2.5 µM) in IMDM/5% FCS on days 5 to 7 of DC generation, DCs were washed and matured in IMDM/5% FCS containing 60,000 scraped *C. albicans* hyphae per well in the presence of 500 U/ml recombinant human GM-CSF (Schering-Plough B.V., Brussels, Belgium), with or without the presence of 600,000 neutrophils (both hyphae and neutrophils had a similar ratio to the surface area as in the 96-well plate). After 48 h, DCs were harvested and stained with a fixable viability dye eFluor 780 (eBioscience, San Diego, CA, USA), and surface marker or intracellular indoleamine-2,3-dioxygenase (IDO) expression was determined by flow cytometry.

### Flow Cytometry

Cells were restimulated for the determination of cytokine production by T cells as described previously (9). On the same day as the restimulation (days 11–13 after the start of culture), cells were stained for CD25 and CD127, and subsequently, cells were fixated and permeabilized with a Transcription Factor Buffer Set (BD Biosciences, San Jose, CA, USA) and stained for FoxP3. A total of 10,000 cells were acquired in the live gate on a FACSCanto machine (BD Biosciences). Alternatively, cells were stained for CD25, CD39, CD49b, CD69, CD127, ICOS, PD1, TIGIT, and TIM3 prior to fixing + permeabilization with the Transcription Factor Buffer Set and staining for CTLA4 and FoxP3. Cells were stained for GARP after 24 h of stimulation with αCD3 and αCD28. A total of 20,000 cells were acquired in the live gate on the SP6800 Spectral Analyzer (Sony, San Jose, CA, USA). The following antibodies were used: αCD3-FITC, αCD4-APC, αCD45RA-FITC, αIFNγ-FITC, αCD25-FITC, αCD39-BV510, αCD49b-BV605, αLAG-3-PE-CF594, αCD69-APC-R700, αPD1-BB515, αTIGIT-BB700, αTIM3-BV480, αGARP-BV750, and αICOSL-PE-CF954 (all from BD Biosciences); αCD127-PE, αCTLA4-PE-Cy5, αCD11c-APC, αCD83-PE-Cy5, αCD86-APC, αHLA-DR-PerCP, and αHLA-DR-BV421 (all from BD Pharmingen, San Diego, CA, USA); αFoxP3-AF647, αCD25-PE-Cy7, αICOS-BV421, and αCD66b-PE (all from Biolegend, San Diego, CA, USA); αCD45RO-PE (DAKO); αIL-17A-eFluor660 (eBioscience); and αIDO-PE (R&D Systems). Data were analyzed using FlowJo™ software (for Windows, Version 10.6.2., Ashland, OR, USA). Heatmaps were generated using Tercen™ (Waterford, Ireland). Cells stained for CD25 and CD127 were sorted with the 4-laser FACSAria IIu SORP (BD Biosciences) prior to restimulation for the assessment of IL-17 expression.

### Cytokine Production Analysis by ELISA

DCs were primed with VD3 as done for the co-cultures, and subsequently, 30,000 DCs were matured in flat-bottom 96-wells with 10,000 *C. albicans* hyphae per well and 30,000 CD40 ligand-expressing murine plasmacytoma cells (J558 cells; a gift from Dr. P. Lane, University of Birmingham, Birmingham, United Kingdom), with or without 60,000 neutrophils (similar ratio as the T-cell co-culture). Sandwich ELISAs were performed on 24-h culture supernatants to determine the concentrations of CXCL8 (Invitrogen), IL-1β (U-CyTech, Utrecht, The Netherlands), IL-10 (BD Biosciences), IL-12_p70_ (own culture), and IL-23 (U-CyTech). For the analysis of IL-12_p70_, 1,000 U/ml of IFN-γ (U-CyTech) was added to the cultures and TGF-β ELISA of eBioscience was used. Without additional DC stimulation, values were below the detection level (20 pg/ml) for IL-12_p70_. Briefly, after blocking overnight the antibody-coated plates in PBS/0.1% Tween 20/1% BSA (PTB), they were incubated with supernatants for 1 h at RT diluted in PTB, and washing was done in PBS/0.1% Tween 20 (PT). Detection antibody with biotin was added for 1 h at RT, and after washing, poly-streptavidin-horseradish peroxidase conjugate (Sanquin) diluted in PTB with 2% Protifar (Nutricia, Utrecht, The Netherlands) was added and incubated for 30 min at RT. Finally, the plates were washed with PT and developed with 3,3′,5,5′-tetramethylbenzidine (TMB, Merck, Germany). The conversion was stopped by adding 1 M of H_2_SO_4_. The absorbance was measured at 450 nm with reference at 655 nm using VersaMax™ (Molecular Devices, Wokingham, UK).

### Statistics

Statistical analyses were performed using GraphPad Prism software (La Jolla, CA, USA, version 8.3.0 for Windows). Repeated-measures one-way ANOVA with Holm–Sidak’s multiple comparisons test was used. The Friedman test with Dunn’s multiple comparisons test, a non-parametric test, was applied if the distribution of data was not normal. The Shapiro–Wilk test was performed to test the normality of data. All statistics used were indicated in the figures and legends. *p*-values of 0.05 or less were considered significant.

## Results

### DC Priming With VD3 Hampers Neutrophil-Induced Th17 Cell Development

**Figure 1:**
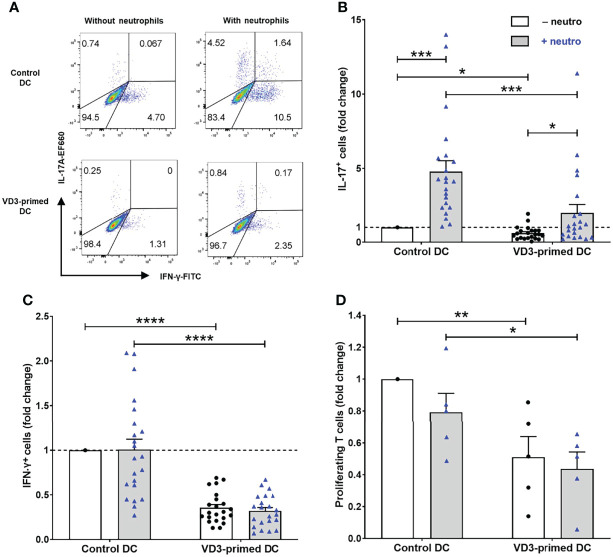
Priming of dendritic cells (DCs) with vitamin D3 (VD3) impedes neutrophil-dependent Th17 cell development. **(A)** IL-17A and IFN-γ expression is shown in restimulated T cells cultured with autologous control or VD3-primed DCs, in the presence or absence of neutrophils. Representative flow cytometry plots are shown. **(B, C)** The percentage of IL-17^+^ cells and IFN-γ^+^ cells normalized to control DCs without neutrophils from 22 independent experiments (mean ± SEM) is depicted. Friedman tests with Dunn’s *post hoc* test were performed. **(D)** T-cell proliferation measured on day 5 normalized to control DCs without neutrophils (mean ± SEM); *n* = 5 independent experiments. One-way ANOVA with Holm–Sidak’s *post hoc* test was performed. *p < 0.05, **p < 0.01, ***p < 0.001, ****p < 0.0001.

Since the effect of VD3 on neutrophil-assisted DC-driven Th17 cell development from human naive T cells is unknown, we investigated the effect of VD3 priming of DCs on T-cell development from naive T cells. In the absence of neutrophils, Th17 cell differentiation was negligible, whereas neutrophil presence elicited the capacity of control DCs to promote Th17 cell differentiation (**Figures 1A, B**), as published before (9). Priming of DCs with VD3 significantly impedes Th17 cell development (**Figure 1B**), with a mean of 6.1% IL-17^+^ cells driven by control DCs versus 1.7% by VD3-primed DCs in the presence of neutrophils. Additionally, Th1 cell development was reduced by VD3 priming of DCs (**Figure 1C**), which is in line with the expectation as VD3 is known to suppress IL-12 expression (23, 24). The potency of VD3 to restrict Th1 cell development was neutrophil-independent.

Furthermore, we observed that the proliferation of T cells cultured with VD3-primed DCs was reduced. Therefore, we assessed the T-cell stimulatory capacity of DCs on day 5 of the culture (**Figure 1D**). VD3 priming of DCs inhibited DC-driven proliferation of naive CD4^+^ T cells (mean control DCs vs. mean VD3-primed DCs without neutrophils: 39% vs. 21%). Moreover, in most donors, we observed that neutrophil addition slightly reduced the proliferation of T cells, although this was not significant either in control DCs or VD3-primed DCs. In the absence of DCs, neutrophils cultured with *C. albicans* hyphae did not stimulate naive T-cell proliferation, as shown in our previous work (9). Taken together, VD3 priming of DCs reduced the capability of DCs to stimulate T-cell proliferation and restricted Th17 and Th1 cell development.

### VD3 Priming of DCs Induces Tregs, Even Under Th17 Cell-Polarizing Conditions

**Figure 2:**
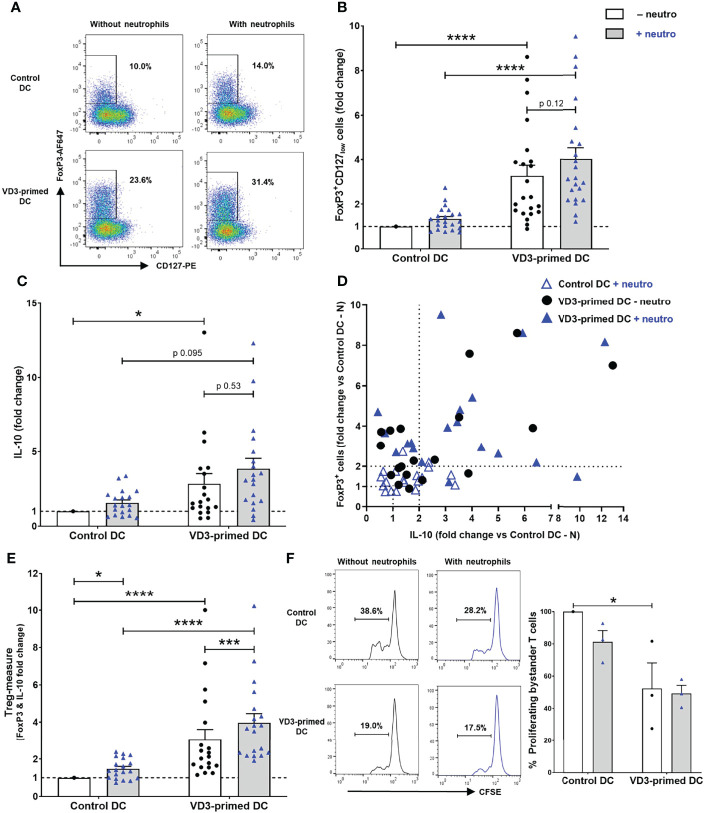
Treg development induction by VD3-primed DCs is increased by the presence of neutrophils. **(A)** Representative flow cytometry plots of FoxP3 and CD127 expression in CD4^+^ T cells cultured with control or VD3-primed DCs in the presence or absence of autologous neutrophils are depicted. **(B)** The percentage of FoxP3^+^CD127_low_ cells normalized to control DCs without neutrophils from 22 independent experiments is shown. **(C)** IL-10 production by T cells is shown relative to control DCs without neutrophils (*n* = 19). **(D)** The induction of FoxP3^+^ cells (*y*-axis) and IL-10 secretion (*x*-axis) normalized to control DCs without neutrophils is depicted per condition. **(E)** Fold change of FoxP3^+^ and IL-10-producing T cells versus control DCs without neutrophils is combined as a Treg measure. **(F)** The suppressive capacity of T cells cultured with control or VD3-primed DCs with or without neutrophils. Results are representative (left panel) or the mean ± SEM of the percentage of proliferating bystander cells relative to control DCs without neutrophils of three independent experiments (right panel). Bar graphs show mean ± SEM. Friedman tests with Dunn’s *post hoc* test were performed in **(B, C, F)**, and one-way ANOVA with Holm–Sidak’s *post hoc* test was performed in **(E)**. *p < 0.05, ***p < 0.001, ****p < 0.0001.

Since we observed a strong reduction in T-cell stimulatory capacity by VD3 priming of DCs (**Figure 1D**), we hypothesized that increased development of Tregs in these conditions could underlie this observation. VD3 is well known to endow DCs with tolerance-inducing properties, resulting in elevated Treg development (11, 14–16). VD3 priming of DCs significantly increased the development of FoxP3^+^CD127_low_CD25^+^ cells, as shown in representative flow cytometry plots (**Figure 2A**) and as combined data from 22 independent experiments and donors (**Figure 2B**). FoxP3^+^CD127_low_ cells were always CD25^+^ (**Supplementary Figure 2A**). Interestingly, we observed a trend of increased Treg development in the presence of neutrophils; however, this effect was not significant. In these Th17-polarizing conditions, VD3 priming of DCs significantly heightened FoxP3^+^ Treg development.

In addition to the distinction of Tregs by FoxP3, CD127, and CD25 expression, IL-10 production is characteristic for various Treg types, including type 1 regulatory (Tr1) cells, ICOS^+^ Tregs, and CD69^+^FoxP3^+^ Tregs, and IL-10 has been proven crucial for their suppressive capacity (14, 19, 20, 34). The combined data of 19 donors showed a significant induction of IL-10 production upon VD3 priming of DCs, also under Th17-polarizing conditions (**Figure 2C**). Again, as seen for FoxP3^+^CD127_low_ T cells, a slight increase in IL-10-producing T cells was observed in the presence of autologous neutrophils. Although FoxP3^+^ Tregs can produce IL-10, not necessarily both FoxP3^+^ Tregs and IL-10-producing T cells are induced simultaneously, as shown by van der Aar et al. (14). In some donors, an increase of FoxP3^+^CD127_low_ Tregs was observed, while in other donors under similar circumstances, IL-10-producing cells were primarily induced (**Figure 2D**). When we further analyzed Treg induction of at least two-fold in the presence of neutrophils, VD3 priming of DCs resulted in the polarization to either FoxP3^+^ Tregs (32% of donors), both FoxP3^+^ and IL-10-secreting T cells (58% of donors), or IL-10-producing T cells (10% of donors). When the induction of FoxP3^+^ and IL-10-secreting T cells was pooled as a Treg measure, neutrophils significantly enhanced Treg development driven by either control DCs or VD3-primed DCs (**Figure 2E**).

Most importantly, we tested the capacity of induced T cells to inhibit the proliferation of autologous bystander-activated CD4^+^ T cells, the ultimate feature of Tregs. T cells cultured with control DCs and neutrophils slightly suppressed the proliferation of bystander-activated T cells compared to T cells without neutrophils (**Figure 2F**). The proliferation of bystander T cells was significantly inhibited by T cells polarized by VD3-primed DCs compared to control DCs, and this was not altered by neutrophil presence. Collectively, the results show that DC-driven T-cell polarization is shifted from pro-inflammatory Th17 cell development toward Treg development upon exposure of DCs to VD3, even in inflammatory conditions where neutrophils are present.

Since we observed increased induction of Tregs under Th17-polarizing conditions (neutrophil presence), we wondered whether these Tregs were identical to the IL-17^+^ subpopulation of T cells. We sorted resting T cells by flow cytometry on the expression of CD25 and CD127, which allowed us to gate for Tregs (CD25^high^CD127_low_ cells) versus presumed T effector cells (Teff; CD25_low_CD127^high^ cells) using the flow cytometry gating strategy in **Supplementary Figure 2B**, prior to restimulation of T cells for the analysis of IL-17 expression (**Supplementary Figure 2C**). In the CD25_low_CD127^high^ T effector cell population, most IL-17^+^ cells were observed in control DCs with neutrophils when compared to the CD25^high^CD127_low_ population of Tregs. Albeit the percentage of CD25^high^CD127_low_ cells was increased in VD3-primed DCs with neutrophil conditions, Th17 cell development was low, as seen in **Figure 1B** and **Supplementary Figure 2C**. Taken together, the CD25^high^CD127_low_ Treg subsets induced by VD3 priming of DCs in the presence of neutrophils are functional suppressor cells with low IL-17 expression.

### Various Treg Subsets Are Induced by VD3-Primed DCs Independent of Neutrophil Presence

**Figure 3:**
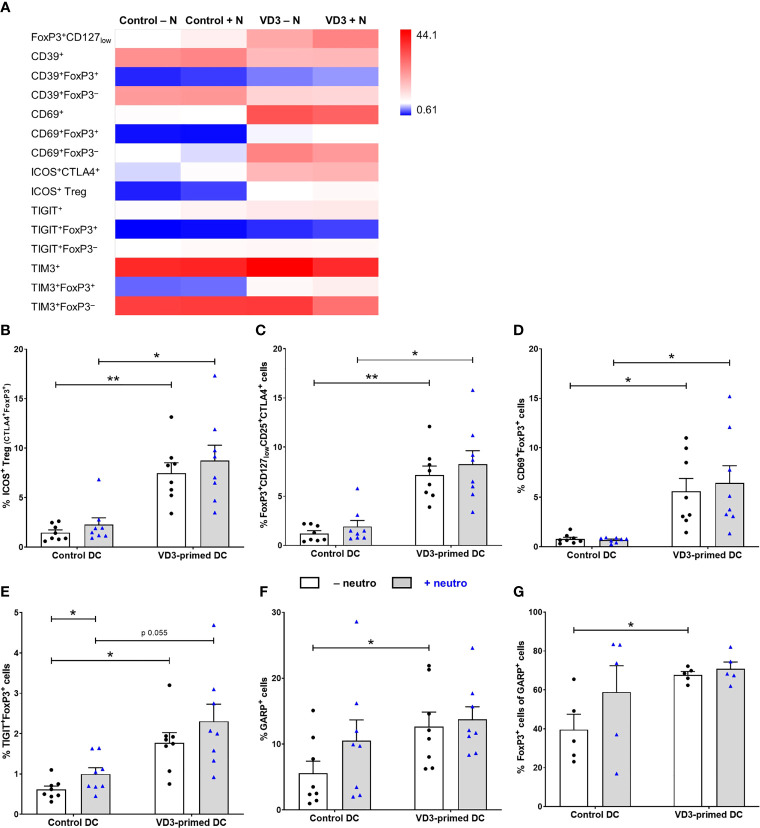
Tregs generated by VD3-primed DCs express functional markers even in the presence of neutrophils. **(A)** Heatmap showing the mean frequency of indicated cell populations gated on single cells of T cells measured on day 11 of control or VD3-primed DC conditions in the absence or presence of neutrophils of eight independent experiments. **(B)** The percentage of ICOS^+^ Tregs is depicted, expressing CTLA4 and FoxP3 in addition to ICOS (*n* = 8). **(C)** The percentage of FoxP3^+^CD127_low_CD25^+^CTLA4^+^ T cells is shown. **(D)** The percentage of CD69^+^FoxP3^+^ T cells is depicted. **(E)** The percentage of TIGIT^+^FoxP3^+^ T cells is shown. **(F)** The percentage of GARP^+^ T cells is depicted after 24 h of restimulation with αCD3 and αCD28 (*n* = 8). **(G)** The percentage of FoxP3^+^ cells in GARP^+^ T cells is shown (*n* = 5). Bar graphs show mean ± SEM. One-way ANOVAs with Holm–Sidak’s *post hoc* test were performed. *p < 0.05, **p < 0.01.

Since different subsets of Tregs, characterized by the expression of specific molecules, have been described, we investigated the expression of functional and Treg subset markers (**Figure 3**). Immune checkpoint cytotoxic T-lymphocyte-associated protein (CTLA)-4 was expressed on both ICOS^+^ Tregs and on natural or induced FoxP3^+^CD127_low_CD25^+^ Tregs (19). The frequency of ICOS^+^ Tregs (ICOS^+^CTLA4^+^FoxP3^+^) and FoxP3^+^CD127_low_CD25^+^CTLA4^+^ Tregs was significantly increased by VD3 priming of DCs, also in the presence of neutrophils (**Figures 3A–C**). Moreover, C-type lectin receptor CD69 and T-cell immunoglobulin and ITIM domain (TIGIT) expression was elevated both on FoxP3^+^ and FoxP3^–^ cells by VD3 priming, while the expression of ectonucleotidase CD39 and T-cell immunoglobulin and mucin domain 3 (TIM3) was specifically induced on FoxP3^+^ T cells and reduced on FoxP3^–^ T cells (**Figure 3A**). In line with the increased polarization toward functional suppressive Tregs, CD69^+^FoxP3^+^ Tregs (**Figure 3D**) and TIGIT^+^FoxP3^+^ cells (**Figure 3E**) were significantly induced by VD3 priming of DCs, even in the presence of neutrophils, while no additional effect of neutrophils was seen. While the average frequency of CD39^+^FoxP3^+^ and TIM3^+^FoxP3^+^ T cells was elevated in VD3-primed DCs, enhanced expression was not seen in all donors and the effect was not significant (**Supplementary Figures 3A–C**).

Furthermore, we measured the expression of glycoprotein A repetitions predominant (GARP), involved in Treg function and homeostasis *via* TGF-β (35), after αCD3 and αCD28 restimulation, since we could not detect GARP expression on day 11 of T-cell culture without restimulation. GARP expression was significantly elevated on T cells polarized by VD3-primed DCs, and the presence of neutrophils did not affect this (**Figure 3F**). The percentage of GARP^+^ T cells that express FoxP3 was significantly increased by VD3 priming as well (**Figure 3G**). We were not able to assess the frequency of the IL-10-producing Tr1 subset, since lymphocyte-activation protein 3 (LAG-3) staining was low, while LAG-3 and CD49b are the most specific Tr1 markers (36). CD49b expression was not affected by VD3 priming or neutrophil presence (**Supplementary Figure 3D**). Overall, these data suggest that under Th17 cell-polarizing conditions (presence of neutrophils), DCs primed with VD3 are still capable of inducing a functional Treg response, with the polarization of naive T cells toward suppressor cells that express a variety of Treg markers, including CD69, ICOS, CTLA4, TIGIT, and GARP.

### VD3 Priming Reduces CD83 Expression and Th1/Th17-Polarizing Cytokine Release by DCs

**Figure 4:**
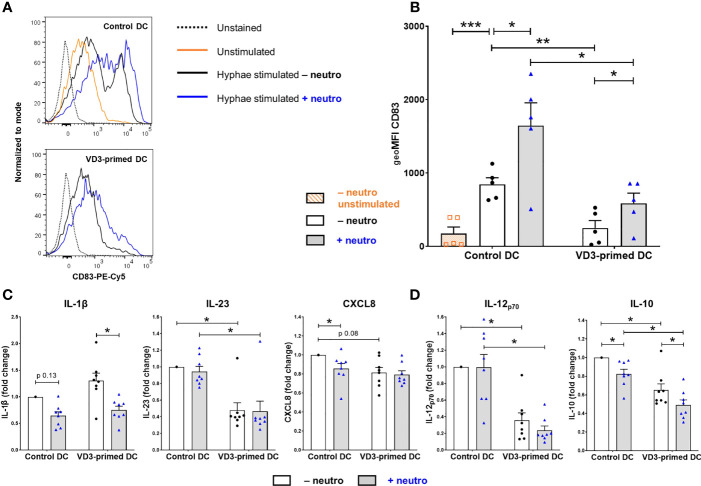
DCs primed with VD3 show reduced CD83 expression and suppressed Th1/Th17-polarizing cytokine production. **(A)** CD83 expression on control or VD3-primed DCs is depicted, either unstimulated or matured with *C. albicans* hyphae in the presence or absence of neutrophils. Representative histograms are shown. **(B)** CD83 data are expressed as geometric mean fluorescence intensity (geoMFI) of five independent experiments (mean ± SEM). **(C)** Cytokine release by control or VD3-primed DCs stimulated with *C. albicans* hyphae and CD40 ligand-expressing cells. Fold change versus control DC conditions without neutrophils is shown (*n* = 8, mean ± SEM). **(D)** IL-12_p70_ and IL-10 secretion by control or VD3-primed DCs is shown in the absence or presence of neutrophils. One-way ANOVAs with Holm–Sidak’s *post hoc* test were performed in **(B, D)**, and Friedman tests with Dunn’s *post hoc* test in **(C)**. *p < 0.05, **p < 0.01, ***p < 0.001.

To understand the mechanism of altered T-cell development, we examined whether VD3 priming and the presence of neutrophils influenced the expression of cell surface molecules associated with DC maturation and the production of key cytokines in response to *C. albicans* hyphae. We used a viability dye to exclude dead cells (both DCs and neutrophils). Ninety percent of neutrophils cultured with *C. albicans* hyphae died overnight (**Supplementary Figure 4A**). VD3 priming of DCs inhibited the induction of CD83 expression by *C. albicans* hyphae stimulation of DCs (**Figures 4A, B**). Furthermore, neutrophils enhanced the expression of CD83 on both control and VD3-primed DCs. This is in line with the ability of lactoferrin, a neutrophil granule protein, to upregulate the expression of CD83 on immature DCs (37, 38). Furthermore, lactoferrin was shown to induce CD86 and HLA-DR expression (38). However, we did not observe enhanced expression of CD86 or HLA-DR by neutrophils nor an effect by the 2-h VD3 priming of DCs (**Supplementary Figure 4B**).

Next, we examined IL-1β and IL-23 release by DCs, known to be involved in the induction and maintenance of a Th17 cell phenotype (6–8). Since *C. albicans* hyphae alone did not induce substantial cytokine release by DCs, costimulation with CD40L was applied, mimicking DC–T-cell interactions, although TGF-β levels in the supernatant were still below the detection level. As expected, VD3 priming inhibited the release of IL-23 versus control DCs without neutrophils (mean ± SD = 122 ± 74 ng/ml), while IL-1β secretion (control DC – neutro: mean ± SD = 0.38 ± 0.31 ng/ml) was not affected by VD3 (**Figure 4C**). To determine whether the reduction in IL-23 was involved in the reduced Th17 cell development observed upon VD3 priming, we added recombinant IL-23 and found a 1.5-fold increase in Th17 cell development in three out of seven donors, a slight induction in three donors, and a reduction in one donor (**Supplementary Figure 5**). Overall, no significant effect of IL-23 was found. Neutrophils did not influence IL-23 secretion, while a reduction of IL-1β release was observed in the presence of neutrophils, which was significant in VD3-primed DCs. Moreover, CXCL8 (77), of which a processed form is required for Th17 cell development from naive CD4^+^ T cells (9), was slightly reduced by VD3 priming of DCs, albeit not significantly (**Figure 4C**). Neutrophils significantly reduced CXCL8 release from DCs in control DCs (control DC – neutro: mean ± SD = 0.64 ± 0.26 µg/ml).

The 2-h VD3 priming of DCs strongly inhibited the secretion of IL-12_p70_ (control DC – neutro: mean ± SD = 6.98 ± 3.76 ng/ml; **Figure 4D**), as expected since the vitamin D receptor (VDR) downregulates both the expression of p35 and p40 subunits (24). Neutrophils reduced IL-12_p70_ release by VD3-primed DCs in each donor, although the effect is not significant. Intriguingly, although the generation of DCs in the presence of VD3 was repeatedly shown to induce IL-10 production in DCs (15, 22), we observed an inhibition in IL-10 release by DCs (control DC – neutro: mean ± SD = 65.68 ± 39.14 ng/ml; **Figure 4D**) that were treated with VD3 for only 2 h prior to *C. albicans* stimulation. Moreover, the presence of neutrophils reduced IL-10 secretion in both control and VD3-primed DCs. Taken together, the 2-h VD3 priming of DCs significantly reduced the secretion of specific T-cell-polarizing cytokines, while neutrophils lowered the release of IL-1β, CXCL8, and IL-10.

### Neutrophils Increase the Expression of IDO and ICOSL in DCs

**Figure 5:**
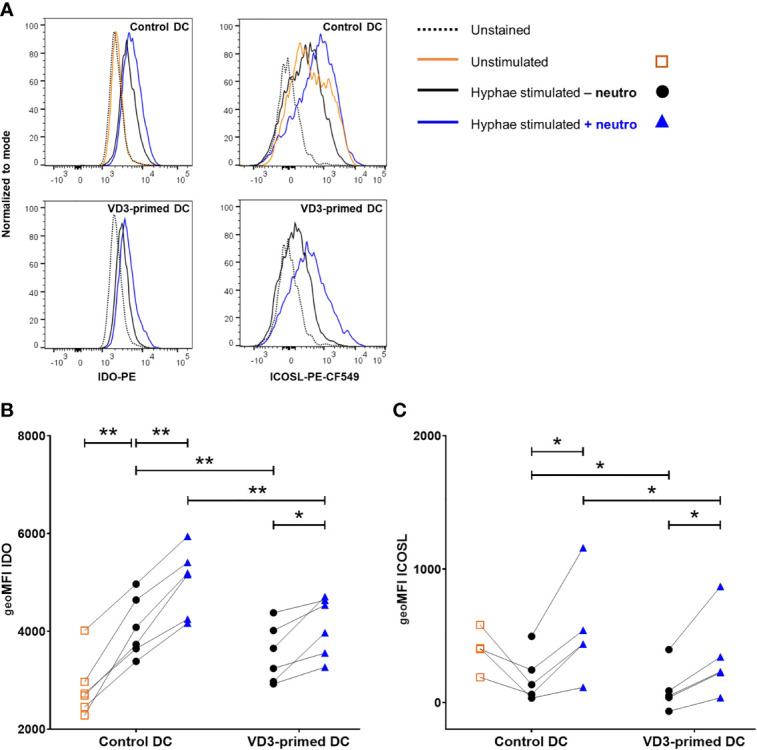
Indoleamine-2,3-dioxygenase (IDO) and inducible costimulatory ligand (ICOSL) expression in DCs is increased by neutrophils. **(A)** Intracellular IDO expression and ICOSL surface expression on control or VD3-primed DCs are shown, either unstimulated or stimulated with *C. albicans* hyphae in the presence or absence of neutrophils. Representative histograms are depicted. **(B)** Intracellular IDO expression is shown as geoMFI (*n* = 6). **(C)** ICOSL on DCs is depicted as geoMFI (*n* = 5). One-way ANOVAs with Holm–Sidak’s *post hoc* test were performed. *p < 0.05, **p < 0.01.

One of the mechanisms *via* which DCs induce Tregs is *via* the activity of the immunosuppressive enzyme IDO, which degrades tryptophan. Downstream metabolites of tryptophan suppress T effector cell responses (39). We investigated whether VD3 priming and neutrophil presence affected the intracellular expression of IDO in DCs. Stimulation with *C. albicans* hyphae significantly increased the expression of IDO compared to unstimulated DCs (**Figures 5A, B**). VD3 priming reduced IDO expression, while neutrophils increased the expression in both control and VD3-primed DCs (**Figures 5A, B**). Consequently, we wondered whether this enhanced IDO expression in DCs could be involved in the increased Treg development by neutrophil presence. Inhibition of IDO was achieved by pretreatment of DCs with epacadostat, an IDO selective inhibitor (39), prior to culture with naive T cells and neutrophils. IDO inhibition did not significantly affect Treg development (data not shown).

In addition to IDO expression in DCs, ICOS ligand (ICOSL) expression is a tolerogenic feature that DCs could employ in order to induce Tregs (20, 40). Therefore, we assessed the expression of ICOSL on control and VD3-primed DCs after stimulation with *C. albicans* hyphae in the absence or presence of neutrophils. Neutrophils significantly increased the expression of ICOSL on DCs and VD3 priming of DCs reduced the expression of ICOSL (**Figures 5A, C**). Hence, both of these tolerogenic DC features of IDO and ICOSL expression follow a similar pattern, with reduced IDO and ICOSL expression in VD3-primed conditions, while neutrophils enhance the expression.

## Discussion

In this study, we show that neutrophil-dependent induction of DC-driven Th17 cell development from naive T cells is impeded by exposure of DCs to VD3, whereas Treg development is increased. Strikingly, neutrophils further enhance Treg development, with a donor-dependent increase in either FoxP3^+^ Tregs, IL-10-producing Tregs, or both major Treg subsets together. The production of the inflammatory cytokines IL-23 and IL-12_p70_ is reduced as well by VD3 priming. Importantly, we observe these clear tolerogenic effects by only the 2-h priming of immature DCs with VD3, rather than exposing DCs to VD3 during maturation, and even under Th17-polarizing conditions (in the presence of neutrophils). Currently, VD3 is used in clinical trials as an immunomodulatory agent to *ex-vivo* generate antigen-specific tolerogenic DCs from patient-derived DC progenitors for subsequent reinfusion into patients (41, 42). However, since this therapy is laborious and expensive, there is a clinical demand for new treatment approaches, such as potentially targeting DCs *in-vivo* (10, 42). The implication of our data substantiates the potential use of VD3 in DC-targeting vaccines, which can be loaded with disease-specific antigens (42), to induce peripheral tolerance in autoimmune diseases or other chronic inflammatory disorders. A distorted Th17/Treg balance characterizes several autoimmune diseases, including rheumatoid arthritis and psoriasis (1, 2). These patients may benefit from a DC-targeting vaccine containing VD3 as an adjuvant since VD3 could tip the balance of T-cell polarization by DCs in the presence of neutrophils from pro-inflammatory Th17 cells to tolerogenic Tregs.

Our data show that DC-driven Th17 cell development from naive human CD4^+^ T cells, which requires the presence of autologous neutrophils (9), is diminished by priming DCs with VD3. As shown in our previous study, donor-to-donor variation exists in Th17 cell induction by neutrophils (9). This could depend on the ability of neutrophils to become activated in co-culture to secrete neutrophil elastase (9), among others, given that neutrophil activation in response to diverse stimuli varies between healthy donors as well (43). Our observations are in line with two studies demonstrating reduced Th17 cell development by VD3-treated DCs. In one study, DCs were cultured with memory CD4^+^ T cells, and in the other study, relatively impure naive CD4^+^ T cells were used (93% naive vs. 98% in our study) (28, 44), which likely resulted in robust Th17 cell development due to memory T-cell contamination (9). We showed a significant reduction in IL-23 release from VD3-primed DCs and hypothesized that this could at least partially underlie the reduced DC-driven Th17 cell development from naive CD4^+^ T cells, given that IL-23 is a Th17 cell-polarizing factor (2). However, Th17 cell development in VD3-primed DCs was not significantly increased by the addition of IL-23 in conditions with neutrophils, demonstrating that reduced IL-23 was not solely responsible for the diminished Th17 cell development.

We excluded a potential role for IL-1β secretion, another Th17 cell-inducing cytokine (2), since surprisingly this cytokine was slightly induced by VD3 priming, albeit not significantly. Moreover, the slight reduction in CXCL8 release from DCs by VD3 priming was not significant and the release of CXCL8 was still high (up to 1 µg/ml) in VD3-primed DCs. Hence, it is not plausible that a reduction in available CXCL8, which could be cleaved by neutrophil elastase to a short form required for Th17 cell development (9), underlies the impeded Th17 cell development by VD3-primed DCs. The reduction in IL-12_p70_ secretion and the corresponding Th1 cell development we observed were in line with previous studies (17, 22–25, 45). Moreover, it was shown that IL-12 potently altered the polarization of T cells cultured with TGF-β away from FoxP3^+^ Tregs toward Th1 cells (46, 47). Therefore, suppression of IL-12 release induced by VD3, either in the presence or absence of neutrophils, appeared to be a prerequisite for Treg development. To our surprise, IL-10 release was significantly suppressed by VD3 priming and by the presence of neutrophils, while IL-10 is known as a powerful anti-inflammatory cytokine that is induced by VD3 (15, 18, 21). However, the study by Sommer et al. compared the effect of exposure to VD3 during differentiation and stimulation or only during stimulation of monocyte-derived DCs. While IL-10 release upon stimulation was indeed increased by VD3-differentiated DCs, control-differentiated DCs stimulated in the presence of VD3 produced less IL-10 when compared to those without VD3 (21). Another study reported reduced IL-10 release by DCs cultured with VD3 and dexamethasone, a known combination for the generation of tolerogenic DCs, while these DCs did exert tolerogenic functions (45). These data corroborate our findings and suggest that IL-10 production is not a prerequisite for the tolerogenic function of DCs.

Furthermore, we assessed the expression of functional and subset-defining Treg makers and whether these were affected by VD3 priming and neutrophil presence. CD39 hydrolyzes extracellular ATP, thereby generating immunosuppressive adenosine, and CD39^+^FoxP3^+^ T cells suppress IL-17 production (48). A direct enhancing effect of VD3 on CD39 expression has been shown on human CD4^+^ T cells (49), while effects *via* DCs were not studied before. CD39 expression on T cells was reduced by VD3 priming of DCs and not affected by neutrophils, and the majority of CD39^+^ cells were negative for FoxP3. In addition to CD39, we assessed the expression of the functional Treg marker CD69. CD69 was initially characterized as an early activation marker of T cells and was considered a marker for tissue-resident immune cells (50). CD4^+^ T cells require CD69 for efficient differentiation to FoxP3^+^ Tregs, and in a steady state, about 50% of Tregs in lymphoid organs express CD69 (50). CD69^+^FoxP3^+^ Tregs secrete elevated levels of IL-10 and are more suppressive in comparison to CD69^−^Foxp3^+^ cells (34, 50). We show that the 2-h VD3 priming of DCs upregulated CD69 expression on T cells, and CD69^+^FoxP3^+^ T cells were significantly enhanced, also in the presence of neutrophils. CD69 promotes activation of the JAK3/STAT5 pathway, which inhibits Th17 cell differentiation (34, 51), possibly providing a mechanistic link between CD69 expression and a shift from Th17 cells to Tregs, which we observed in the presence of neutrophils. Additionally, we found that the frequency of TIGIT^+^FoxP3^+^ cells was significantly higher by VD3 priming of DCs, while TIM3^+^FoxP3^+^ cells were increased as well, albeit not significantly. TIGIT and TIM3 expression on Tregs are both associated with specific suppression of pathogenic Th1 and Th17 cell responses (52, 53). Taken together, we identified both highly suppressive Tregs and populations known to specifically suppress Th1 and Th17 cell responses.

A limitation of this study is that we were not able to identify the Tr1 population of Tregs since we could not measure distinct LAG-3 expression, even after restimulation with αCD3 and αCD28 for 3 days. LAG-3 and CD49b are the most specific markers to characterize the IL-10-producing subset of Tr1 cells (36). We could detect CD49b expression on >20% of T cells on day 11 of culture without restimulation, and no differences were observed between culture conditions. Moreover, IL-10-producing T cells were significantly increased by VD3 priming of DCs. We show an increased frequency of other IL-10-producing Treg types by VD3 priming, which like Tr1 cells rely on IL-10 production for their suppressive function (19, 20, 34), including CD69^+^FoxP3^+^ T cells and ICOS^+^ Tregs. The reduced expression of ICOSL by VD3 priming on *C. albicans* matured DCs does not correlate with the increased frequency of the ICOS^+^ Treg subset. ICOS^+^ Tregs produce large amounts of IL-10 (5–8 ng/ml in ELISA), on average six-fold more IL-10 than ICOS^–^ Tregs (54). T cells generated by VD3-primed DCs in the presence of neutrophils produced 3.4 ng/ml of IL-10 on average after restimulation. The ICOS^+^ Treg subset (average frequency 8.7% in VD3 DCs vs. 2.3% in control DCs with neutrophils) may account for the increased IL-10 production by T cells cultured with VD3-primed DCs.

VD3 priming reduced the expression of CD83 and the immunosuppressive enzyme IDO in DCs, while the expression of both was increased by neutrophils. The question remains whether direct cell contact of neutrophils and DCs is required for this increased expression. We observed by immunohistochemistry staining that neutrophils and DCs interact (data not shown), but it remains to be assessed whether this direct contact or rather soluble factors mediate the neutrophil-induced change in DC phenotype. The VD3-mediated effect of impaired upregulation of CD83, classically a maturation marker of DCs, has been shown before in lipopolysaccharide-stimulated human DCs differentiated in the presence of VD3 (23). In contrast to our data, it was previously shown that murine DCs generated from precursor cells in the presence of VD3 have elevated IDO expression (55). However, the regulation of IDO expression in human DCs might be different. IDO expression in human DCs requires non-canonical NF-κB signaling (56). The suppressive effect of VD3 on NF-κB activity has mainly been described on canonical pathway components (57). Nevertheless, VD3 was shown to inhibit RelB transcription in a DC-derived cell line, and RelB is a transcription factor of the non-canonical NF-κB pathway (58, 59). Therefore, we hypothesize that the observed inhibition in IDO expression in DCs by 2-h priming with VD3 could be due to suppressed non-canonical NF-κB signaling.

Despite the intriguing observation that autologous neutrophils increase Treg development, especially by VD3-primed DCs, we were not able to pinpoint the mechanism *via* which neutrophils enhance Treg induction without encountering VD3 themselves. Neutrophil elastase was shown to induce the production of TGF-β in human DCs *in vitro*, which favored polarization toward FoxP3^+^ Tregs (10, 60, 61). Furthermore, neutrophils themselves produce and release TGF-β (62, 63). Myeloperoxidase, another neutrophil granule component, was shown to suppress human DC activation and IL-12 production (10, 64). In the presence of neutrophils, IL-12 release by VD3-primed DCs was further suppressed, although not significantly, which could be involved in the enhanced Treg induction since restricted IL-12 release is a prerequisite for Treg development. We observed that 90% of neutrophils died upon overnight culture with *C. albicans* hyphae, and it is likely that apoptotic neutrophils are engulfed by DCs in our co-culture system (65). The process of neutrophil clearance by DCs was considered critical to the maintenance of peripheral tolerance (66), and apoptotic neutrophils were shown to inhibit IL-12 production by DCs (65). Moreover, co-culture of DCs with apoptotic cells increased IDO expression in DCs (67), indicating that the process of neutrophil clearance could also play a role in the increased IDO expression by neutrophils.

Taken together, our data show that even in the presence of neutrophils, VD3 priming of DCs induces the development of both FoxP3^+^ and IL-10-producing Tregs, including ICOS^+^ Tregs, while Th17 cell development is impeded. This finding may be beneficial for patients suffering from chronic inflammatory disorders in which neutrophils infiltrate affected tissues and are associated with disease progression, including neutrophilic asthma, rheumatoid arthritis, and systemic lupus erythematosus (3, 30, 31). Furthermore, we show that CD69^+^FoxP3^+^ and TIGIT^+^FoxP3^+^ Treg subsets are induced by VD3-primed DCs, which are especially beneficial for the targeted suppression of pathogenic Th17 cell responses that contribute to disease progression in psoriasis and rheumatoid arthritis, among others (1, 2). Collectively, these data support the potential use of VD3 as an adjuvant in DC-targeting vaccines for the treatment of chronic inflammatory disorders.

## Data Availability Statement

The original contributions presented in the study are included in the article/**Supplementary Material**. Further inquiries can be directed to the corresponding author.

## Ethics Statement

The studies involving human participants were reviewed and approved by the Institutional Review Board of the Amsterdam University Medical Center (METC 2015_074). The participants provided their written informed consent to participate in this study.

## Author Contributions

FH, TG, and EJ designed the research. FH, ET-K, and TC performed the research. FH analyzed the data and wrote the manuscript. TG and EJ revised the manuscript. All authors contributed to the article and approved the submitted version.

## Funding

This work was supported by Amsterdam UMC, University of Amsterdam.

## Conflict of Interest

The authors declare that the research was conducted in the absence of any commercial or financial relationships that could be construed as a potential conflict of interest.

## Publisher’s Note

All claims expressed in this article are solely those of the authors and do not necessarily represent those of their affiliated organizations, or those of the publisher, the editors and the reviewers. Any product that may be evaluated in this article, or claim that may be made by its manufacturer, is not guaranteed or endorsed by the publisher.
