# Supplementary material for: Vitamin D3 Priming of Dendritic Cells Shifts Human Neutrophil-Dependent Th17 Cell Development to Regulatory T Cells
Source: Front Immunol. 2022 Jul 7;13:872665. doi: 10.3389/fimmu.2022.872665 (PMC9301463; doi:10.3389/fimmu.2022.872665)
Supplement: Supplementary file 1 [file DataSheet_1.pdf]

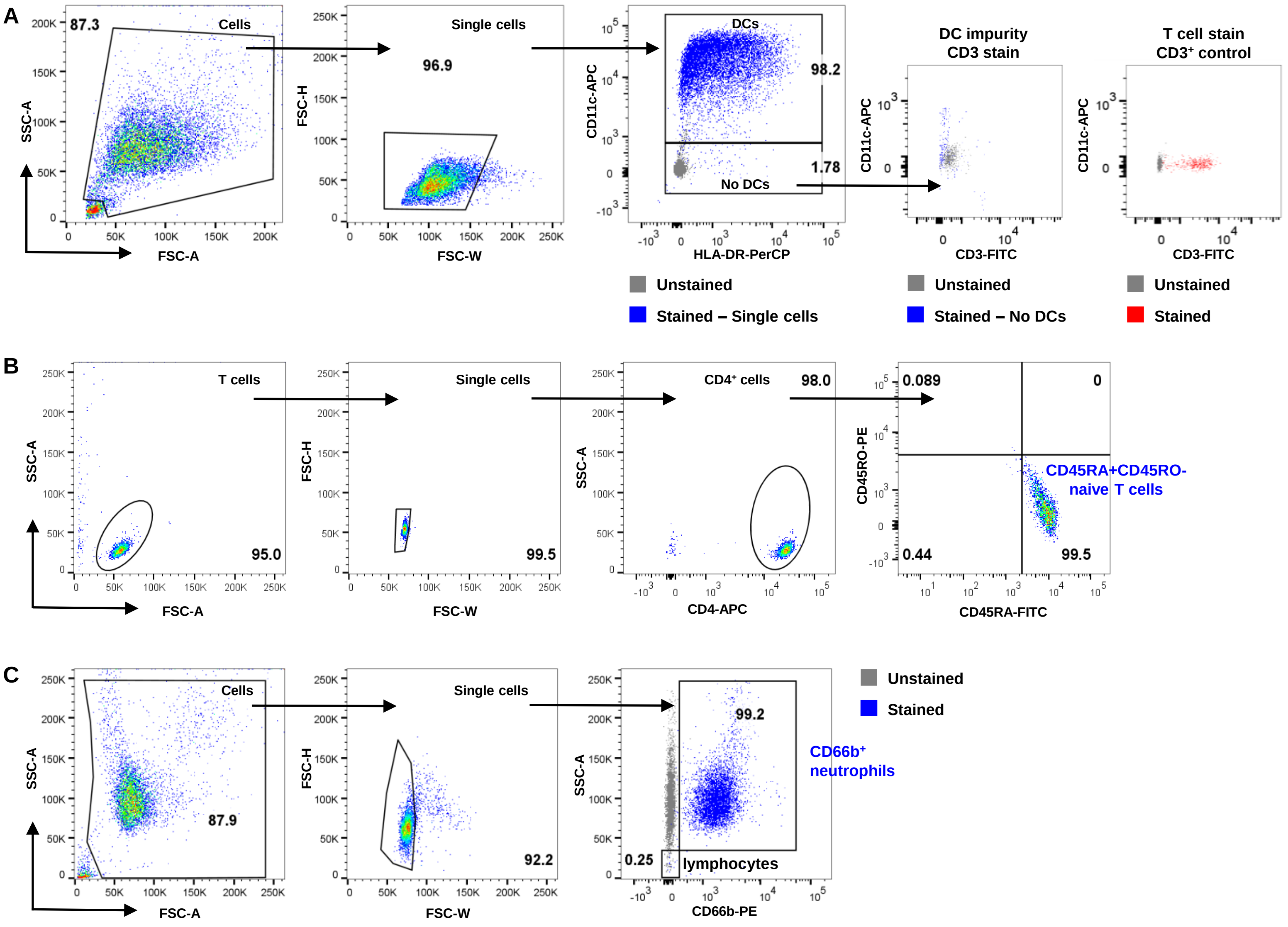

SUPPLEMENTARY FIGURE 1 | Purity of generated moDCs, isolated naive T cells and neutrophils. **(A)** Gating strategy is shown of moDCs stained for CD11c, HLA-DR and CD3 to assess purity. CD11c$^+$ cells are gated as ‘DCs’ (98.2% of single cells) and CD11c$^-$ cells as ‘No DCs’ (1.78%). The majority of ‘No DCs’ were CD3$^-$ cells and as positive control for the CD3 antibody T cells were stained (red population). **(B)** Purity of isolated CD4$^+$CD45RA$^+$CD45RO$^-$ cells, naive T cells, is depicted (99.5% of CD4$^+$ cells). **(C)** Purity of CD66b$^+$ neutrophils (99.2% of single cells) is shown after isolation from fresh blood. Cells with low granularity (low SSC) are CD66b- and are distinguished as lymphocytes based on SSC$_{low}$CD66b$^-$.

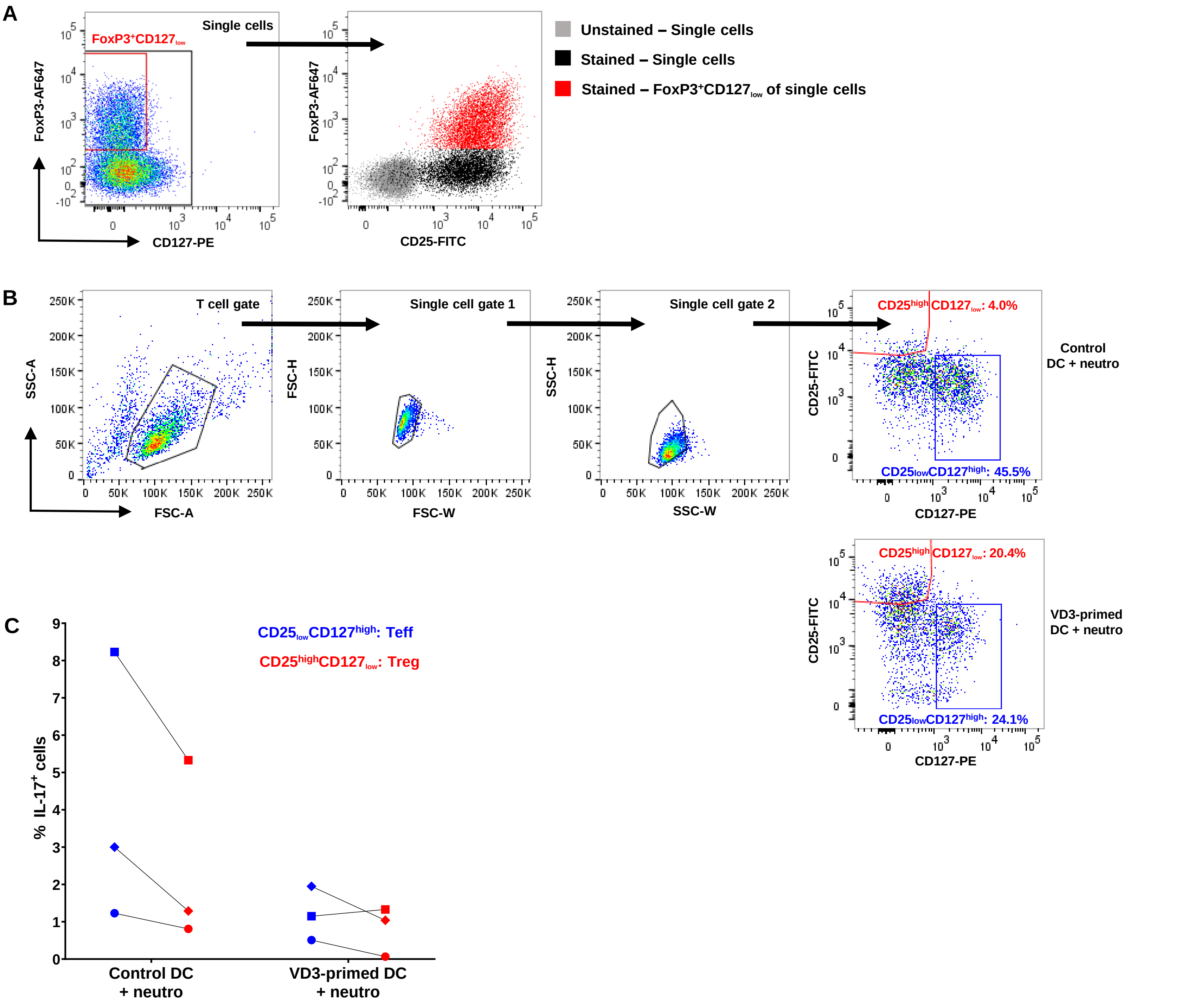

SUPPLEMENTARY FIGURE 2 | IL-17^{+} cells are not identical to CD25^{high}CD127$_{low}$ Treg subset. **(A)** Representative flow cytometry staining plots for FoxP3 versus CD127 or versus CD25 are depicted of condition VD3-primed DCs with neutrophils (n = 13). Cells of different gated populations are shown in the right plot; single cells of an unstained sample (grey), single cells of a stained sample (blue) and the FoxP3^{+}CD127$_{low}$ cells of the gate in the left plot (red). **(B)** Gating strategy used for sorting CD25$_{low}$CD127^{high} cells (Teff cells) and CD25^{high}CD127^{low} (Tregs) is depicted for control DCs without neutrophils and a CD25 x CD127 plot is also shown for VD3-primed DCs with neutrophils. **(C)** Percentage of IL-17^{+} cells of 3 independent experiments is shown in CD25$_{low}$CD127^{high} (blue) versus CD25^{high}CD127$_{low}$ (red) cells in control DC and VD3-primed DC conditions with neutrophils, each donor a different symbol.

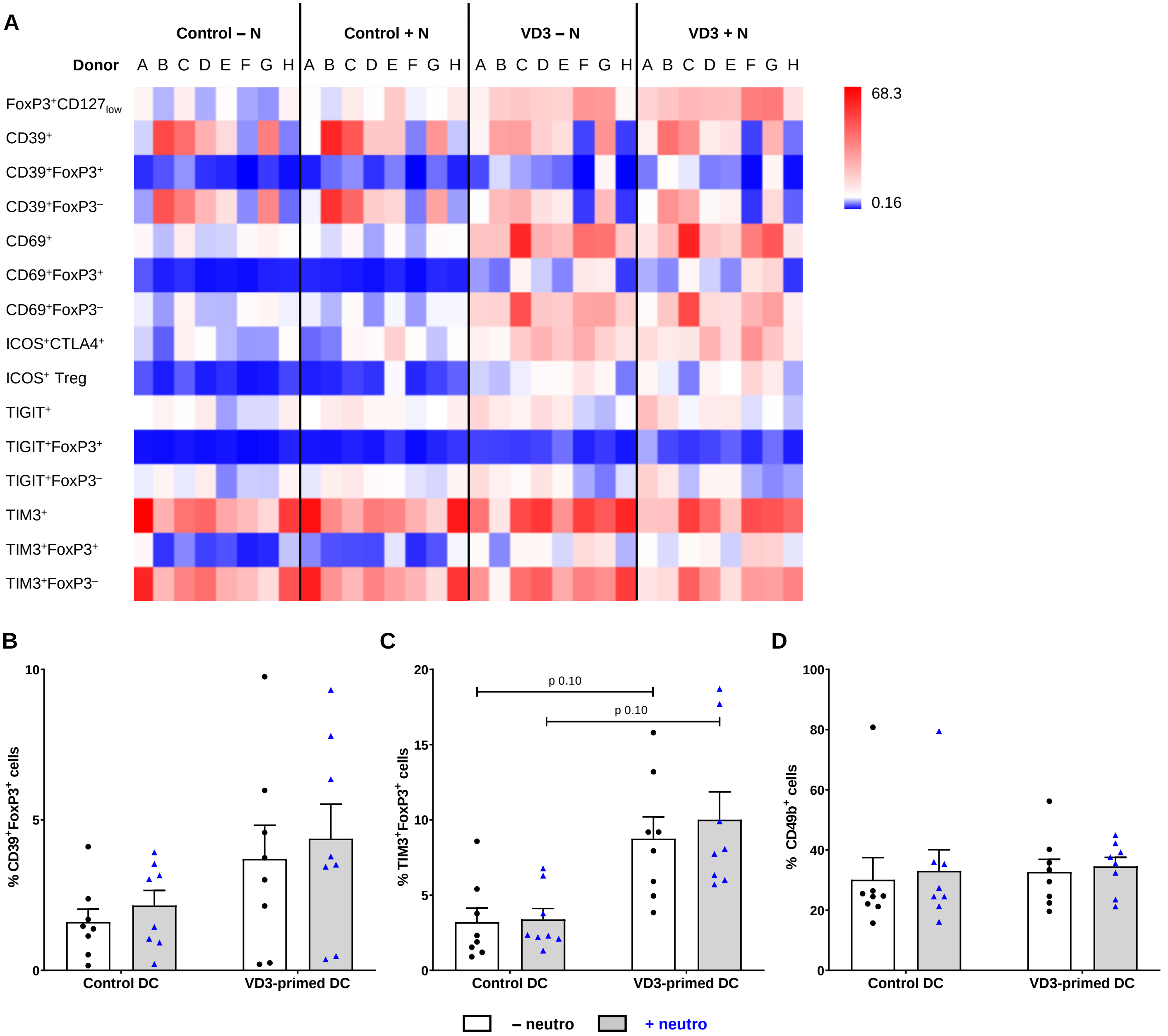

SUPPLEMENTARY FIGURE 3 | VD3-priming of DCs increases Treg marker expression and percentage of FoxP3+ T cells of GARP+ T cells. **(A)** Heatmap showing frequency of indicated cell populations of eight independent donors gated on single cells of T cells measured at day 11 of control or VD3-primed DC conditions in absence or presence of neutrophils. **(B)** Percentage of CD39+FoxP3+ T cells is depicted (n = 8). **(C)** Percentage of TIM3+Foxp3+ T cells is shown (n = 8). **(D)** Percentage of CD49b+ cells (Tr1 subset) is shown at day 11 of culture (n = 8); Friedman test with Dunn's *post-hoc* test. In B and C one-way ANOVA with Holm-Sidak's *post-hoc* test was performed. Bar graphs show mean ± SEM. All p values were > 0.05.

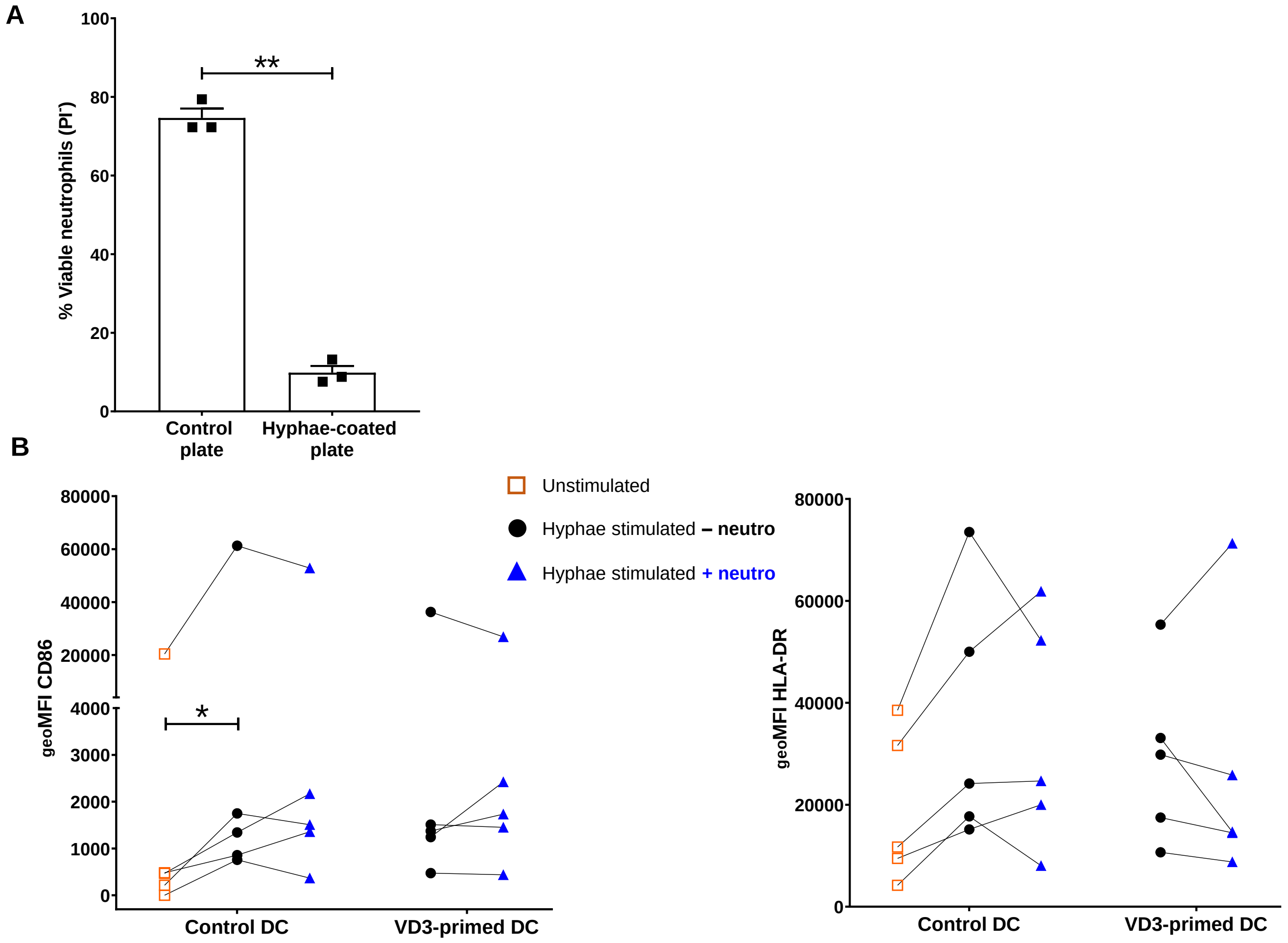

SUPPLEMENTARY FIGURE 4 | CD86 or HLA-DR expression on DCs unaffected by VD3-priming or neutrophil presence. **(A)** Neutrophils were cultured for 16 h on either a control 96-well flat-bottom plate or a hyphae-coated plate at 100,000 cells/well in IMDM with 5% HI-HS. Non-adherent neutrophils were collected by washing and stained with propidium iodide (PI) purchased from Sigma-Aldrich. Neutrophils negative for PI by flow cytometry assessment are depicted of three donors. Bars indicate mean ± SEM and a paired t-test was done. **(B)** CD86 and HLA-DR expression on control or VD3-primed DCs is depicted, either unstimulated or matured with C albicans hyphae in presence or absence of neutrophils. Data are expressed as geoMFI of n = 5. Friedman test with Dunn's *post-hoc* test was performed for CD86, and one-way ANOVA with Holm-Sidak's *post-hoc* test for HLA-DR. * $p < 0.05$, ** $p < 0.01$.

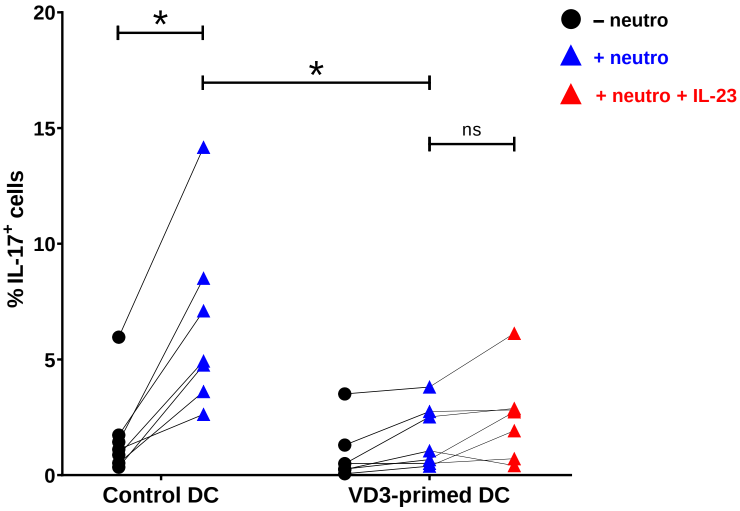

SUPPLEMENTARY FIGURE 5 | IL-23 alone is not able to rescue Th17 cell development by VD3-primed DCs. Percentage of IL-17$^+$ cells in restimulated T cells cultured with control or VD3-primed DCs, in presence or absence of neutrophils. Additionally, recombinant human IL-23 (30 ng/mL) was added to VD3-primed DCs with neutrophils. Data of 7 independent experiments are depicted; Friedman test with Dunn's *post-hoc* test. * $p < 0.05$.
